# Integrating molecular tools into leishmaniosis surveillance: evaluation of a commercial qPCR kit in sand fly vectors

**DOI:** 10.1186/s13071-026-07440-y

**Published:** 2026-05-29

**Authors:** Blanca Dehesa-García, Henar Alonso, Imanol Ruiz-Zarzuela, Cristina Cervera-Acedo, David Martínez-Durán, Sarah Delacour, Juan Antonio Castillo, José A. Oteo, Francisco Collantes, María Paz Peris, Ignacio Ruiz-Arrondo

**Affiliations:** 1 Department of Animal Pathology, Faculty of Veterinary Sciences, University of Zaragoza, Zaragoza, Spain; 2 Health Research Institute Aragón, Zaragoza, Spain; 3 Department of Microbiology, Paediatrics, Radiology, and Public Health, Faculty of Medicine, University of Zaragoza, Zaragoza, Spain; 4 Instituto Agroalimentario de Aragón (IA2), Universidad de Zaragoza-CITA, Zaragoza, Spain; 5 Center of Rickettsiosis and Arthropod-Borne Diseases (CRETAV), Center for Biomedical Research of La Rioja (CIBIR), San Pedro University Hospital, Logroño, Spain; 6 Departamento de Zoología y Antropología Física, Facultad de Biología, Universidad de Murcia, Murcia, Spain

**Keywords:** Diagnostic validation, *Leishmania infantum*, Phlebotomine, qPCR, Xenomonitoring

## Abstract

**Background:**

Leishmaniosis is a zoonotic disease caused by protozoan parasites of the *Leishmania* genus, primarily transmitted through the bites of infected female phlebotomine sand flies. *Leishmania infantum* is the most prevalent *Leishmania* species in the Mediterranean Region, affecting both humans and animals, mainly dogs. Integrated surveillance strategies, including xenomonitoring, are essential for early detection and cost-effective control in endemic areas. In 2021, an increase in human leishmaniosis cases was reported in the Region of Murcia (Spain), prompting an intensified entomological surveillance effort aimed at assessing *Leishmania* prevalence in vector populations. Phlebotomine sand flies were collected and analysed to determine the circulation of *Leishmania* spp. in the area. Subsequently, these field-collected samples were used to evaluate the diagnostic performance of a commercial real-time PCR (qPCR) kit designed for the detection of *Leishmania* DNA.

**Methods:**

A comparative–retrospective analysis was carried out at the Parasitology Laboratory of the Department of Animal Pathology, University of Zaragoza. A total of 314 DNA extracts from pooled female sand fly samples, collected in urban and peri-urban areas of 29 municipalities in the Region of Murcia near recently reported human leishmaniosis cases (2021–2022), were analysed. Sampling was conducted in June and September 2023 using CO_2_-baited light traps to investigate the presence of *Leishmania* spp. in sand flies. The performance of the VIASURE qPCR assay (CerTest Biotec, Zaragoza, Spain) was assessed against a structured reference method consisting of an in-house qPCR assay, conventional PCR followed by Sanger sequencing and a SYBR Green qPCR used to resolve discordant results.

**Results:**

Of the 314 pooled samples analysed, 21 tested positive for *Leishmania* spp. using the reference methods, while the VIASURE assay identified 23 *Leishmania* spp.-positive pooled samples. After resolving discrepancies, the obtained analytical sensitivity and specificity were 0.95 (95% confidence interval [CI] 0.76–0.99) and 0.99 (95% CI 0.97–0.99), respectively.

**Conclusions:**

The commercial VIASURE qPCR assay showed high concordance with established molecular methods and demonstrated reliable performance for detecting *Leishmania* DNA in sand fly vectors. To our knowledge, this is the first study to specifically validate a commercially available qPCR kit in phlebotomine sand flies. These findings support its potential utility in entomological surveillance programs and public health interventions in leishmaniosis-endemic regions.

**Graphical abstract:**

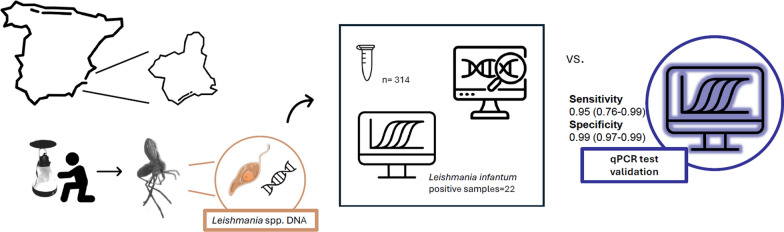

## Background

Leishmaniosis is a zoonotic disease caused by infection with parasitic protozoans of the *Leishmania* genus. More than 20 *Leishmania* species have been identified as being pathogenic, with *Leishmania infantum* being the most widespread of these [1]. Transmission occurs mainly through the bites of *Leishmania* spp.-infected female phlebotomine sand flies. In 2023, the WHO reported an estimated global annual incidence of 700,000–1 million new cases [2], butt only a small proportion of infected individuals ultimately develop symptomatic disease. The major risk factors are primarily associated with conditions that facilitate the reproduction, growth and development of sand flies as well as exposure to the sand flies. Such conditions include—but are not limited to— poor housing and sanitary conditions, migratory movements and environmental and climatic changes that favor the geographical spread and increased density of sand flies [3]. In addition, it has been noted in recent years that the presence of competent reservoirs is necessary for an active circulation of the pathogen, sustaining the parasite's transmission cycle.

*Leishmania infantum*, which causes human (both visceral and cutaneous) and canine leishmaniosis, is considered to be endemic in the Mediterranean region [4]. Across Europe, the prevalence of human leishmaniosis increased between 2005 and 2020, particularly due to the expansion of suitable climatic conditions for vectors. With this expansion, leishmaniosis is currently an endemic zoonosis in Spain, with its prevalence varying by geographical region [5]. The main sand fly species associated with *Leishmania* transmission in Spain are *Phlebotomus perniciosus* and *P. ariasi*, with an activity period that ranges from May to October [6]. In response to its epidemiological relevance, leishmaniosis has become a notifiable human disease in Spain since 2015 [5].

Integrated surveillance is crucial for monitoring and controlling vector-borne diseases in endemic areas. The detection of active circulation of the pathogen through xenomonitoring allows for more cost-effective interventions [3]. For this reason, a sampling effort of sand flies was conducted from June to September 2023, near the residences of the human leishmaniosis cases in the Region of Murcia, where a rapid increase in incidence had been reported since 2021.

The detection of *Leishmania* in phlebotomine sand flies is essential for understanding transmission dynamics and guiding control measures. In the study reported here, we evaluated a commercial real-time PCR (qPCR) diagnostic kit, originally developed for human diagnosis, to assess its suitability for testing sand fly samples and its potential application in entomological surveillance. Published validations of qPCR assays for *Leishmania* spp. detection in vector samples are limited. To this end, the results obtained with the kit were compared with those from reference molecular methods using previously characterized phlebotomine pools to directly estimate the diagnostic performance of the commercial qPCR assay.

In addition to analytical validation, potential operational advantages, such as protocol harmonization across laboratories and shorter hands-on time compared with some in-house assays, were also considered. Because the assay targets *Leishmania* spp., it may support detection of multiple species and, if transferable to vector matrices, could offer flexible and cost-effective testing for surveillance. The implementation of such an assay was considered to be a potential aid for decision-making within an established vector control programme.

## Methods

### Study design

The present study was designed as a comparative–retrospective observational study to assess the performance of the molecular assay under study at the Parasitology Laboratory of the Department of Animal Pathology at the Faculty of Veterinary Sciences, University of Zaragoza (Spain).

### Study samples

Female sand flies captured in the Region of Murcia (Spain) from May to September 2023 were included in the study. The study period was divided into three sub-periods (May, June and September) based on the activity of the main vector species in Spain, *Phlebotomus perniciosus.* During these periods, two standardized sampling rounds were conducted at 25 fixed sampling points in urban and peri-urban areas. Targeted collections focused on locations near the residences of previously reported human leishmaniosis cases (2021–2022), as well as a variety of other environments, including livestock farms, dog shelters, caves and additional habitats. Sand flies were collected using paired BG-Pro light traps (LT) baited with CO_2_ and BG-Lure traps (one trap equipped with white light [WL] and the other with ultraviolet light [UV] (all Biogents AG, Regensburg, Germany) as well as castor oil sticky traps (ST). Light traps operated for 24 h, while sticky traps remained in place for 4 days to minimize DNA degradation and oil interference during extraction. Additionally, sand flies were occasionally collected during parallel mosquito surveillance at 17 supplementary locations, covering a total of 29 municipalities. Specimens were identified to species level at the Faculty of Biology, University of Murcia (Spain). Female sand flies were morphologically classified based on head and genitalia structures; remaining body parts were preserved at − 80 °C in Dulbecco’s modified Eagle’s medium (DMEM) for LT collections and in absolute ethanol for ST collections. Pools consisting of 1–10 female individuals were formed based on trap location, collection date, sex and the species of sand fly. Each pool was treated as a single sample, resulting in a total of 314 pools included in the study for the validation of the diagnostic kit. Samples were kept at − 80 °C and transported to the Center of Rickettsiosis and Arthropod-Borne Diseases (CRETAV), at San Pedro University Hospital–CIBIR in Logroño (Spain), for nucleic acid extraction and *Leishmania* detection.

### Sample preparation and nucleic acid extraction

In the laboratory, samples were thawed and manually homogenized using a lancet. DNA extraction was performed using the DNeasy Blood & Tissue Kit (Qiagen, Hilden, Germany) following the manufacturer’s instructions. DNA was resuspended in 90 µl of elution buffer and stored at − 80 °C until molecular evaluation.

An aliquot of DNA from each pool was sent at − 20 °C to the Parasitology Laboratory of the Department of Animal Pathology of the Faculty of Veterinary Sciences of the University of Zaragoza (Spain), for the evaluation of the commercial kit under study.

### Molecular methods for the detection of *Leishmania* spp.

#### Reference PCR assays

A qPCR assay targeting a conserved region of *Leishmania* kinetoplast DNA (kDNA) minicircle [7] was performed with a modification in probe concentration (600 nM). Primers and the TaqMan probe used were LEISH-1 (5′-AACTTTTCTGGTCCTCCGGGTAG-3′), LEISH-2 (5′-ACCCCCAGTTTCCCGCC-3′) and LEISH-P (5′-FAM/AAAAATGGG/ZEN/TGCAGAAAT/3IABkFQ-3′), respectively. Amplifications were performed using the QuantStudio 5 Real Time PCR System (Applied Biosystems, Thermo Fisher Scientific, Waltham, MA, USA) using the NZYSupreme qPCR probe Master Mix (NZYTech, Lisbon, Portugal). The thermal cycling profile was 95 °C for 2 min, 45 cycles at 95 °C for 15 s and 60 °C for 1 min. Each amplification run contained positive (DNA from MCAN/ES/Z002 promastigote culture obtained from a naturally infected dog, as in previously described methods [8]) and negative controls (no template control); all the samples were run in duplicate. A qPCR result was considered positive when reactions showed amplification (cycling threshold [Ct] < 40) in at least one of the two replicates. The results were interpreted using QuantStudio™ Design & Analysis Software v.1.5.1 (Applied Biosystems, Thermo Fisher Scientific).

Conventional PCR targeting a partial region of the rRNA internal transcribed spacer 2 (ITS2) was also performed as previously described [9] using the NZYTaq II 2X Green Master Mix (NZYTech). The primers used were: LGITSF2 (GCATGCCATATTCTCAGTGTC) and LGITSR2 (GGCCAACGCGAAGTTGAATTC). The PCR assay was performed in a T100™ thermal cycler (Bio‐Rad Laboratories, Hercules, CA, USA) using the following thermal cycling profile: 95 °C for 5 min, followed by 40 cycles of 95 °C for 30 s, 60 °C for 30 s and 72 °C for 1 min, with a final extension of 72 °C for 7 min.

Amplicons of the conventional PCR of the expected size were sequenced in both directions using an ABI PRISM® 3130 automated sequencer (Applied Biosystems, Thermo Fisher Scientific). Nucleotide sequences were compared with those available in GenBank using BLAST (MegaBlast option; https://blast.ncbi.nlm.nih.gov/Blast.cgi).

#### Molecular method under study

The commercial assay under study (VIASURE *Leishmania* Real Time PCR Detection Kit; CerTest Biotec, Zaragoza, Spain) is a qPCR monoplex kit targeting a conserved region of the 18S ribosomal RNA (rRNA) gene from *Leishmania* spp. using specific primers and fluorescent-labelled probes. *Leishmania* spp. was detected using a FAM™ fluorophore-labelled probe, and the internal control for monitoring PCR reaction inhibition targeting synthetic DNA included in the reaction mix was detected using a HEX™ fluorophore-labelled probe. Positive and negative controls (no template control) were included in each run. Each reaction was performed in a final volume of 20 µl containing 15 µl of Master Mix and 5 µl of DNA template.

The thermal cycling protocol consists of 1 cycle at 95 °C for 2 min and 45 cycles at 95 °C for 10 s and 60 °C for 50 s. Fluorescence data are collected during the annealing and extension step at 60 °C. Amplification was performed using the CFX Connect Real-Time PCR Detection System (Bio-Rad Laboratories), and the results were analysed using the same system's software (Bio-Rad CFX Manager, Version 2.1). The results of the amplification were interpreted following the manufacturer’s instructions: a sample was considered to be *Leishmania* spp.-positive when it displayed a typical sigmoidal amplification curve and had a Ct value < 40.

#### Reference diagnosis for discordant sample resolution

A SYBR Green-based qPCR assay that amplifies a fragment of a conserved region of the kDNA minicircle of *Leishmania* spp. was carried out with previously described primers [10] to resolve discordant results. The primers used were Jw11-F (5′-CCTATTTTACACCAACCCCCAGT-3′) and Jw12-R (5′-GGGTAGGGGCGTTCTGCGAAA-3′). All amplifications were conducted using the MiniOpticonTM System (Bio-Rad Laboratories) with an initial incubation at 95 °C for 10 min, followed by 44 cycles of 95 °C for 5 s (denaturation), 55 °C for 40 s (annealing, amplification and acquisition of fluorescence), and the results were analysed using the system's (Bio-Rad CFX Manager, Version 2.1). A sample was considered to be *Leishmania* spp.-positive when the amplification curve crossed the fluorescence threshold within the defined cut-off (Ct < 35).

### Data collection and analysis

The data for sensitivity (SE), specificity (SP), negative predictive values (NPV) and positive predictive values (PPV) were calculated using Freeware software to perform the meta-analysis, MetaDiSc [11]. This calculation was performed using a 95% confidence interval.

A receiver operating characteristic (ROC) curve analysis was generated using IBM SPSS Statistics for Windows, Version 24.0 software (IBM Corp., Armonk, NY, USA), with the area under the curve (AUC) score for the VIASURE assay results calculated. The optimal diagnostic cut-off value was determined by calculating the Youden index of the ROC curve. Differences were considered to be statistically significant when the probabilities of equality *P* values were ≤ 0.05.

### Analysis results acceptance criteria

The clinical SE, SP, PPV and NPV acceptance criterion was a calculated value of at least 80%, with an ideal value of 95%. For the ROC curve analysis, the AUC score and Q* index values should be close to 1, which is the ideal test value, with a calculated value of at least 0.95.

## Results

Morphological analysis of the collected sand flies identified four sand fly species (*P. perniciosus*, *Phlebotomus papatasi*, *Phlebotomus sergenti* and* Sergentomyia minuta*), and *Leishmania* DNA was detected in all of these specimens. Of the 314 pools analysed, 21 tested positive for *Leishmania* spp. DNA by the reference qPCR, with 20 of the latter testing also positive based on the ITS2 PCR assay; nucleotide sequences showed 99.4–100% similarity with *L. infantum* in 12 pools, and the species could not be determined in the remaining eight pools, which were subsequently classified as *Leishmania* spp. Using the commercial assay under study, 23 samples were qPCR-positive for *Leishmania* DNA, and the remaining 291 samples were qPCR-negative, yielding four discordant results compared with the initial diagnosis of three false positive results and one false negative result. Consequently, the third method, consisting of a SYBR green-based real-time PCR assay, was used to resolve these discrepancies (Table 1). Analysis of the four discordant results concluded that the assay under study reported one false negative and one false positive result; the remaining two positive results were corroborated as true positives based on the results of the SYBR green assay.

**Table 1:** Results for the four discordant samples obtained with the reference molecular methods, the VIASURE *Leishmania* Real Time PCR Detection Kit and the assay (SYBR Green-based qPCR targeting kDNA minicircle), and the final classification of each sample for validation of the VIASURE assay

Sample ID	Reference methods^a^	VIASURE assay^b^	SYBR green qPCR^c^	Final classification^d^
1	Positive (Ct = 39.7)	Negative	Positive (Ct = 31.9)	False negative
2	Negative	Positive (Ct = 38.3)	Negative	False positive
3	Negative	Positive (Ct = 36.8)	Positive (Ct = 31.2)	True positive
4	Negative	Positive (Ct = 37.2)	Positive (Ct = 30.3)	True positive

*Ct* Cycle threshold,* kDNA* kinetoplast DNA,* qPCR* quantitative PCR

^a^Reference methods included: (i) probe-based real-time PCR targeting *Leishmania* kDNA minicircles and'(ii) conventional PCR targeting the ITS2 region, followed by Sanger sequencing

^b^Assay under study (VIASURE *Leishmania* Real Time PCR Detection Kit; (CerTest Biotec, Zaragoza, Spain)

^c^SYBR Green-based real-time PCR targeting *Leishmania* kDNA minicircles (assay to resolve discordant results)

^d^The final classification is defined relative to the results of the VIASURE assay (true positive, true negative, false positive, false negative)

The obtained analytical SE and SP of the VIASURE *Leishmania* Real Time PCR Detection Kit were 0.95 (95% confidence interval [CI] 0.76–0.99) and 0.99 (95% CI 0.97–0.99), respectively, and the PPV and NPV were 0.95 (95% CI 0.76–0.99) and 0.99 (95% CI 0.97–0.99), respectively (Table 2). The AUC score obtained in the ROC curve analysis was 0.977, the calculated Youden index for the ROC curve was 0.987 and the cut-off value was < 39.84.

**Table 2:** Contingency table and diagnostic performance of the VIASURE *Leishmania* Real Time PCR Detection Kit for *Leishmania* spp. DNA detection

Reference method results and metrics of VIASURE assay	VIASURE positive	VIASURE negative	Total
*Reference method result*			
Positive	22 pools	1	23
Negative	1 pool	290	291
Total	23 pools	291	314
*Diagnostic accuracy of the VIASURE assay*^a^			
Sensitivity	0.95 (0.76–0.99)		
Specificity	0.99 (0.97–0.99)		
PPV	0.95 (0.76–0.99)		
NPV	0.99 (0.97–0.99)		

*NPV* Negative predictive value,* PPV* positive predictive value

^a^Sensitivity, specificity, PPV and NPV of the VIASURE *Leishmania* Real Time PCR Detection Kit were compared with the metrics of the reference method. Values are presented with 95% confidence intervals in parentheses

## Discussion

Effective epidemiological surveillance of arthropod vectors and the pathogens they may carry and transmit is crucial for accurately assessing risk and implementing timely control measures in a given area. In our study, sand fly collection and analysis in the vicinity of reported human leishmaniosis cases in the Region of Murcia (Spain), an area of increasing leishmaniosis incidence, revealed the circulation of *Leishmania* spp. in *P. perniciosus*, *P. papatasi*, *P. sergenti* and *S. minuta* sand flies. Most of the positive samples were confirmed as *L. infantum*. Sand flies of the genus *Sergentomyia* have historically been assumed to feed only on reptiles and to play no role in the transmission of human leishmaniosis. However, recent studies using molecular methods have determined that *S.* *minuta* not only feed on mammals but can also carry *L. infantum* and *L. major* (both pathogenic species to humans and animals) [11–16]. The presence of these four species in the Region of Murcia is consistent with previous sand fly collection studies performed in 2015 in this area [17].

The results obtained in this study are comparable to, or indeed superior to, those reported in the literature for other qPCR experimental designs targeting the same gene, which reported sensitivity and specificity values of 64% and 100% [18], 98% and 84% [19] and 90.9% and 18.2%, respectively [20]. All of these qPCR designs correspond to in-house or laboratory-developed PCR assays that have been tested in human lesion samples for the diagnosis of cutaneous leishmaniosis or American tegumentary leishmaniosis. Similarly, a study conducted to validate an in-house qPCR with the same gene target across both canine and human tissue samples showed sensitivity and specificity values of 96.6% and 98.5%, respectively, for canine samples and 89.47% and 98.7%, respectively, for human samples [21]. On the other hand, the prototype Leish-qPCR, which also targets the same gene, was validated using human samples with clinical suspicion of visceral or cutaneous leishmaniosis, yielding sensitivity and specificity values of 97.7% and 98.1%, respectively [22]. Finally, previous validation of the assay under study using skin biopsy and visceral human clinical samples reported sensitivity and specificity values of 81.8% and 100%, respectively [23]. While the current results are an improvement on those previously obtained, it is important to note that the sample type employed in this study is different from that used in the previous one. This means that a direct comparison of the results is not possible.

Despite the presence of inconsistencies in the results obtained, it is noteworthy that these inconsistencies exhibited low positivity (high Ct values, see Table 1) when they reported positive results. Notwithstanding the potential disparities in sensitivity and specificity among the diverse molecular methodologies, an alternative hypothesis for the observed variations pertains to the freezing and thawing cycles during DNA extraction.

The ROC-derived threshold (Ct < 39.84) was essentially concordant with the manufacturer’s pre-established cut-off (Ct < 40), supporting the transferability of the clinical decision rule to pooled phlebotomine samples in this setting. Given that ROC-optimized thresholds are sample-dependent and may overfit a specific dataset, maintaining the manufacturer’s whole-number cut-off improves interpretability and comparability across studies and laboratories. Importantly, applying Ct < 40 in our dataset did not materially alter diagnostic performance, suggesting that a shared cut-off for clinical samples and phlebotomine pools is appropriate under the experimental conditions applied.

In the last few years, the highest human leishmaniosis incidence rates in Spain have been reported in the Mediterranean region (Comunidad Valenciana, Balearic Islands and Region of Murcia) [24]. Since 2016 (Annual incidence (AI) = 0.78 cases/100,000 inhabitants), the incidence of leishmaniosis in Spain has followed an increasing trend, with a drop in 2020 as a consequence of the COVID-19 pandemic (AI = 0.58 cases/100,000 inhabitants) and a recovery to pre-pandemic values in 2023 (AI = 0.8 cases/100,000 inhabitants) [25, 26]. Notably, the incidence rate (IR) in the Region of Murcia is increasing more rapidly than the national average [24]. Furthermore, the increase in visceral leishmaniosis in the Region of Murcia appears to be associated with apparently immunocompetent individuals, in contrast to the past when cases were consistently linked to some form of immunodeficiency [27]. These trends highlight the need for integrated vector surveillance to better understand the epidemiological changes observed in leishmaniosis.

However, it is important to note that the study and contextualization of the xenomonitoring samples lack a strong link between *Leishmania* DNA presence in the phlebotomine sand fly population and the occurrence of human leishmaniosis cases in neighbouring areas, such as the identification of animal species in blood meals through cytochrome* b* gene (*cytb*) sequencing [28].

A review of the current literature on commercially available real-time PCR assays targeting *Leishmania* DNA revealed that the present study is the first to consider vector surveillance samples (i.e. phlebotomine sand flies) as a sample type for performance evaluation, despite the consensus on the importance of xenomonitoring in the management of vector-borne diseases such as leishmaniosis [29–32].

The ideal diagnostic method would possess high sensitivity and specificity values, reduced turnaround times and sufficient standardization to minimize variability between laboratories and other sources of methodological bias. This approach would ensure truly comparable results and enable consistent assessments over time and across territories. Moreover, the potential for enhanced throughput and increased sample capacity represents a substantial opportunity in the domain of vector-borne disease surveillance, which is of mounting importance and analytical value.

In-house qPCR analyses require the acquisition of various different reagents for the reaction mix, including separate primers, probes and master mix reagents, many of which require refrigeration or freezing storage. Also, it may be necessary to undertake a phase of troubleshooting to optimize the qPCR protocol. Conversely, a ready-to-use mix typically does not necessitate an optimization stage and involves a reduction in hands-on time and reagent handling, thereby minimizing the risk of contamination. Notwithstanding the aforementioned points, the substantial price increase per reaction may not be viable for smaller laboratories with reduced testing volumes. Additionally, as the assay targets *Leishmania* spp. at the genus level, it identifies *Leishmania* DNA but cannot differentiate species, so species-level identification must be performed separately when required for epidemiology studies.

## Conclusions

The results of the VIASURE assay demonstrated a strong correlation with those of the various reference molecular methods described herein and proved to be a good tool for the detection of *Leishmania* DNA in sand fly samples. This method enables the rapid and accurate identification of infected vectors, which is essential for timely disease control and prevention, as well as a potential unified approach among laboratories engaged in vector surveillance. To our knowledge, this study constitutes the first targeted validation of a commercial qPCR assay in phlebotomine sand flies, establishing an essential reference framework for its potential deployment in entomological surveillance and public health monitoring.

## Data Availability

Partial ITS2 gene sequences generated in this study have been deposited in GenBank under accession numbers PZ091451 (*n* = 1), PZ091452 (*n* = 10, four of which correspond to shorter sequences) and PZ091453 (*n* = 1).
